# Novice teachers’ emotional labor: a study of volunteer Chinese teachers in African Confucius Institutes

**DOI:** 10.3389/fpsyg.2025.1618970

**Published:** 2025-08-19

**Authors:** Mengying Wu, Wenle Yan

**Affiliations:** Research Institute for International and Comparative Education, Shanghai Normal University, Shanghai, China

**Keywords:** emotional rules, emotional labor strategies, volunteer Chinese teachers, Africa, novice teachers

## Abstract

**Introduction:**

This study investigates how volunteer Chinese teachers (VCTs) in African Confucius Institutes navigate emotional labor and examines the emotional rules governing their strategic choices.

**Methods:**

Through semi-structured interviews with 16 VCTs, we identified four categories of emotional rules that shape their emotional labor strategies: professional rules, organizational rules, socio-cultural rules, and personal rules, in descending order of influence.

**Results:**

Professional rules encompass adherence to language teaching guidelines, native cultural teaching norms, and linguistic proficiency requirements, each demanding significant emotional commitment. Organizational rules emphasize hierarchical respect, conflict avoidance, and cultural ambassadorship, particularly through internalized role identification. Socio-cultural rules reflect adaptation to local cultural norms and conformity to local perceptions of Chinese people, demonstrating the dynamic nature of cultural adaptation. Personal rules emerge from individual teaching experiences and student interactions, reflecting teachers’ unique pedagogical approaches. In response to these rules, teachers employ various emotional labor strategies, including surface acting, deep acting, and expressions of naturally felt emotions, through which they apply techniques such as pretending, disguising, and cognitive restructuring. The findings reveal that novice VCTs often experience stress from professional demands and cultural adaptation, frequently managing these challenges through emotional regulation without adequate institutional support.

**Discussion:**

This study extends emotional labor theory by demonstrating how professional, organizational, sociocultural, and personal emotional rules interact in cross-cultural teaching contexts. The findings suggest that emotional rules are not merely external constraints but are actively interpreted and internalized by teachers through their professional practice. These insights provide valuable guidance for developing targeted support systems to enhance VCTs’ emotional well-being and teaching effectiveness in cross-cultural contexts, particularly through pre-departure cultural sensitivity training and ongoing psychological support services.

## 1 Introduction

The teaching profession, recognized for its significant emotional demands (Hargreaves, 2000), has attracted increasing scholarly attention regarding teacher emotional labor (Benesch, 2018; Brown et al., 2023; Nazari and Molana, 2023; Yin et al., 2017). Hochschild’s (1983) foundational work, The Managed Heart, introduced emotional labor (EL) as the regulation of emotions to create appropriate public displays through facial and bodily expressions. This concept highlights the potential conflict between authentic emotional experiences and professionally mandated emotions (Benesch and Prior, 2023). Hochschild (1983) argues that individuals regularly adapt their emotional expression to meet established expectations through “emotional rules” governing acceptable behavior, which significantly influence teachers’ emotional labor strategies (Zhang et al., 2020; Stark and Bettini, 2021). While emotional labor theory posits that organizational demands shape emotion regulation (Grandey, 2000), studies on teachers highlight how institutional and sociocultural factors (e.g., top-down policies, classroom power dynamics) exacerbate this gap (Nazari and Karimpour, 2022). These emotional rules encompass both “feeling rules” for appropriate emotional experiences and “display rules” for their outward expression (Ashforth and Humphrey, 1993; Hochschild, 1983; Stark and Bettini, 2021).

In teaching, emotional labor involves teachers regulating their internal feelings and external expressions to meet professional expectations (Yin and Lee, 2012). Teachers employ three main emotional labor strategies: surface acting (SA), deep acting (DA), and expression of naturally felt emotions (ENFE), a framework well-established in literature (Choi and Kim, 2015; Peng et al., 2019; Yin et al., 2013; Zhu and Zhou, 2022). Beyond mere emotional display, EL integrates with teachers’ professional competencies (Brown et al., 2023). Research links teacher EL to various outcomes including burnout, job satisfaction, and humor styles (Brown et al., 2023; Lee, 2019; Liao et al., 2020; Nazari et al., 2023; Yao et al., 2015). For beginning teachers, the relationship between emotional labor and professional outcomes appears particularly complex. Li (2024a) demonstrated that beginning EFL teachers experience non-linear effects of emotional labor, suggesting that novice VCTs’ inconsistent emotional regulation strategies may be less effective than developing stable patterns of either minimal or intensive emotional management. While deep acting and natural emotional expression typically yield positive outcomes (Asif, 2022; Yin et al., 2013, 2019), excessive surface acting can negatively impact teacher well-being (Tsang et al., 2021).

Language teachers face particularly complex emotional demands due to the heightened affective nature of language learning contexts (Gkonou and Miller, 2019; Schutz and Lee, 2014). They frequently engage in emotional regulation, including suppression and masking of emotions during instruction (Loh and Liew, 2016). Benesch (2019) emphasizes how discursive communities’ emotional protocols govern professional affective displays in language teaching settings. However, research on teacher emotional labor has predominantly focused on English language instruction (Benesch, 2018; Nazari and Karimpour, 2022; Yang et al., 2021; Zhu and Zhou, 2022), leaving Chinese language teachers in international contexts largely unexplored.

The global expansion of Chinese language education, particularly through Confucius Institutes in Africa, presents a unique context for examining emotional labor. Unlike English language teachers who have been extensively studied, VCTs represent an understudied yet significant group in cross-cultural education, facing unique challenges in emotional labor due to their novice status, voluntary nature, and distinctive cultural context. As of March 2022, Africa hosted 62 Confucius Institutes and 48 Confucius Classrooms across 45 countries, serving approximately 150,000 students as of 2017. Volunteer Chinese teachers (VCTs) in African contexts merit particular attention due to their distinctive role combining language instruction, cultural promotion, and international relations, adding complexity to their emotional labor beyond typical language teaching demands. Furthermore, many VCTs begin their overseas teaching careers immediately after completing undergraduate or graduate studies, encountering various professional and emotional challenges as novice teachers. Previous research highlights that novice language teachers often face intensified emotional demands as they transition into professional roles, particularly in constructing and negotiating their professional identity, thus increasing their emotional labor (Kocabaş-Gedik and Ortaçtepe Hart, 2021). Yet, the specific emotional rules guiding VCTs’ emotional labor in these cross-cultural, voluntary, and novice contexts remain largely unexplored.

Understanding the emotional rules governing VCTs’ emotional labor in African Confucius Institutes is crucial for both theoretical advancement and practical application. Theoretically, exploring how emotional rules operate in cross-cultural, voluntary, and novice teaching contexts extends existing emotional labor frameworks (Hochschild, 1983; Stark and Bettini, 2021; Yin and Lee, 2012). Practically, gaining insights into these emotional rules can inform the design of targeted training programs, effective support mechanisms, and proactive institutional policies, empowering volunteer teachers to manage their emotional labor more effectively and sustainably. Understanding how VCTs use emotional labor strategies can ultimately enhance their professional satisfaction, psychological well-being, and pedagogical effectiveness, thereby benefiting both teachers and learners in international educational settings.

Given the unique professional context and emotional challenges confronting VCTs in African Confucius Institutes, it becomes imperative to explore the specific emotional rules they perceive and how these rules shape their use of emotional labor strategies. Thus, this study addresses two central research questions:

RQ1. What emotional rules govern volunteer Chinese language instructors’ behavior in African teaching contexts?

RQ2. How do these emotional rules shape VCTs’ use of emotional labor strategies?

## 2 Theoretical framework

### 2.1 Teacher’s emotional labor and emotional labor strategies

This study is grounded in emotional labor theory, which has evolved significantly since Hochschild’s (1983) seminal work. Within educational contexts, the theoretical framework encompasses three interconnected components: the concept of emotional labor in teaching, emotional rules (including both feeling rules and display rules), and emotional labor strategies (surface acting, deep acting, and naturally felt emotions). This framework provides a comprehensive lens for examining how teachers manage their emotions in professional settings. These three components—emotional labor concept, emotional rules, and emotional labor strategies—interact dynamically in the teaching context. While emotional rules provide the framework for acceptable emotional displays, teachers’ choice of emotional labor strategies is influenced by their understanding and internalization of these rules. The effectiveness of their emotional labor, in turn, shapes how they interpret and adapt to emotional rules. This dynamic interaction is particularly significant in cross-cultural teaching contexts, where teachers must navigate multiple layers of emotional rules while developing their professional competence.

Teaching is fundamentally an emotion-laden profession requiring extensive emotional engagement and management (Hargreaves, 2001; Sutton and Harper, 2009; Winograd, 2003; Yilmaz et al., 2015). Teaching qualifies as emotional labor through three defining elements: direct personal engagement with students, intentional emotional influence on learners, and adherence to professional emotional guidelines (Hochschild, 1983; Winograd, 2003; Yilmaz et al., 2015; Zembylas, 2002). Contemporary understanding of educational emotional labor has evolved beyond Hochschild’s (1983) initial framework, as teachers’ emotional engagement frequently aligns with professional fulfillment and educational care principles (Isenbarger and Zembylas, 2006; Winograd, 2003). Furthermore, educators frequently demonstrate spontaneous emotional adaptability without institutional directives (Oplatka, 2007; Sutton, 2004).

Research has identified three primary emotional labor strategies employed by teachers. Surface acting involves modifying external emotional expressions while maintaining different internal feelings (Grandey, 2000, 2003; Diefendorff et al., 2005). Deep acting encompasses internal emotional recalibration through cognitive reframing processes (Hochschild, 1983; Morris and Feldman, 1996; Basim and Beğenirbaş, 2012). The expression of naturally felt emotions represents teachers’ spontaneous alignment between felt emotions and professional expectations (Ashforth and Humphrey, 1993; Morris and Feldman, 1996).

Studies indicate that teachers typically combine these strategies rather than relying on a single approach (Liu et al., 2013; Mou, 2014; Tian et al., 2009). The effectiveness of these strategies varies across sociocultural contexts (Kitayama et al., 2000; Lee and Van Vlack, 2018) and professional experience levels (Brown et al., 2014; Liu, 2007). Research shows positive relationships between naturally felt emotions and deep acting strategies, while surface acting often leads to professional burnout (Cukur, 2009; Hülsheger et al., 2010).

### 2.2 Emotional rules in cross-cultural teaching contexts

Language teachers, as emotional laborers (Benesch, 2017), face distinct challenges in cross-cultural settings (Norton and De Costa, 2018; Schutz and Lee, 2014). Cultural contexts significantly shape both the expression and interpretation of emotions in educational settings, with contrasting patterns of acceptable emotional expression underscoring the culturally contingent nature of emotional rules (Cheshin, 2020; Winograd, 2003; Morris and King, 2018). Teachers typically revise their emotion management techniques as they navigate through different cultural environments (Bao et al., 2022). Studies of expatriate teachers reveal consistent patterns of emotional masking and adaptation strategies (Morris and King, 2018; Oplatka and El-Kuran, 2022).

Educational institutions establish specific organizational emotional rules that shape teachers’ emotional displays through both formal requirements and informal expectations (Morris and King, 2018). These organizational rules often reflect broader cultural values while adding distinct institutional requirements for emotional expression and management (Oplatka and El-Kuran, 2022). Language teachers encounter diverse emotional rules arising from the unique pedagogical and interpersonal challenges inherent in language learning contexts. For instance, the expectation that foreign language teachers should cultivate a caring and empathetic demeanor, motivating students while simultaneously mitigating language-learning anxiety (Kang, 2022), can lead teachers to suppress their authentic emotional experiences to meet perceived student needs.

Novice teachers face intensified challenges during their professional transition (Jiang et al., 2020), particularly in cross-cultural environments. These challenges encompass managing pedagogical content and classroom dynamics (Chaaban and Du, 2017), while simultaneously navigating unfamiliar institutional emotional rules (Kocabaş-Gedik and Ortaçtepe Hart, 2021). Their professional development involves understanding and implementing appropriate emotional labor strategies within new cultural contexts (Intrator, 2006). Research documents specific emotional labor demands among novice language teachers in international settings, including maintaining supportive learning environments while managing cultural differences (Hulda and Zhao, 2024), adapting to local educational norms (Morris and King, 2018), and developing context-appropriate emotional regulation strategies (Schutz and Lee, 2014).

Volunteer teachers represent a distinct category within cross-cultural language education, facing unique emotional labor challenges that extend beyond those encountered by regular expatriate teachers. Recent research on heritage language volunteer teachers has revealed specific feeling rules that structure their emotional labor, including expectations to “be generous and caring,” “be committed and dedicated,” “be a good and efficient teacher,” and “have no expectations of the community” (Afreen and Norton, 2024). Their temporary status and transitional role identity require them to balance institutional expectations with developmental needs (Chaaban and Du, 2017), while simultaneously navigating complex identity negotiations across multiple roles. Li (2024b) documented how volunteer teachers experienced emotional labor as a catalyst for identity construction across professional, volunteer, and caregiving responsibilities. This perspective is particularly relevant for VCTs who must simultaneously fulfill teaching, cultural ambassadorship, and personal development roles, creating layers of emotional rules that interact in complex ways.

While existing literature has extensively documented emotional labor in various teaching contexts, research specifically addressing non-English language volunteer teachers in cross-cultural settings remains limited. Although studies have examined emotional labor among novice teachers (Nazari et al., 2023) and cross-cultural teaching contexts (Hulda and Zhao, 2024), the intersection of these dimensions—particularly in the context of Chinese language teaching in Africa—represents an understudied area. This gap is especially significant given that these teachers must navigate multiple layers of emotional rules while developing their teaching competence in unfamiliar cultural environments. Understanding these multiple dimensions of emotional rules can inform the development of targeted support mechanisms and training programs for teachers in cross-cultural educational contexts (Kocabaş-Gedik and Ortaçtepe Hart, 2021).

## 3 Methodology

A qualitative research design was implemented to investigate the visible and underlying emotional guidelines that shape VCTs’ experiences. Semi-structured interviews were selected as the primary data collection method, as they enable researchers to thoroughly examine complex social phenomena (Feagin et al., 2016). While Chinese language instruction in Africa emerged in the middle of the twentieth century, it experienced substantial growth in the early 1960s, driven by strengthening diplomatic bonds and increased student mobility between China and African nations. The teaching of Chinese language to African students initially took place predominantly in China. However, this educational paradigm changed substantially with two key developments: the establishment of the Confucius Institute program in 2004, and the subsequent launch of Africa’s pioneer Confucius Institute at Kenya’s University of Nairobi in 2005. The subsequent proliferation of Confucius Institutes and Classrooms across Africa has significantly broadened access to Chinese language education. Since 2020, strengthening China-Africa relations and increasing demand have sustained high numbers of dispatched VCTs. These teachers not only provide instruction in universities, primary and secondary schools, and Confucius Institutes/Classrooms, but also participate in cultural exchange activities, fostering mutual understanding and friendship. The majority of these volunteers are master’s degree holders or recent bachelor’s graduates, often specializing in Chinese international education. The volunteer cohort is predominantly young, typically aged 22–30, with a higher concentration in the 26–30 age range, and a significant gender imbalance favoring female volunteers. Most volunteers have limited prior experience teaching Chinese as a foreign language or living abroad. Volunteer assignments typically last 1 year.

This study involved semi-structured interviews with 16 volunteer Chinese language teachers assigned to various African countries to teach in Confucius Institutes and Classrooms. Six participants are currently teaching in Africa, while 10 have completed their one-year volunteer assignments and returned to China. Participants were recruited through snowball sampling, initiated through contacts made during a pre-departure training program specifically designed for VCTs assigned to Africa. These teachers were posted across the African continent, including countries like Uganda, Botswana, Rwanda, and others, teaching diverse student populations ranging from primary and secondary school pupils to university students and corporate professionals. All 16 teachers contacted agreed to participate in the study. Following institutional ethical approval, participants provided informed consent, acknowledging confidentiality protocols and study commitments. Pseudonyms are used throughout to protect participant anonymity. The sample consisted of early-career teachers, none exceeding 5 years’ experience, with 11 making their first foray into international Chinese language teaching. This enables our data to provide a focused examination of novice teachers’ emotional labor during their overseas teaching assignments. The demographic information of the 16 participants is presented in Table 1.

**Table 1:** Demographic information of research participants (*N* = 16).

Teachers	Gender	Recent degrees	Age range	Years of teaching	Country
AB	F	Masters	26–30	2	Botswana
DL	F	Masters	26–30	1	Rwanda
HW	F	Masters	26–30	2	Uganda
KC	F	Masters	26–30	1	Botswana
LC	F	Masters	26–30	1	Comoros
LM	F	Masters	26–30	less than 1	Mozambique
MA	F	Masters	26–30	1	Mauritius
MB	F	Bachelor	21–25	less than 1	Mauritius
QB	M	Masters	26–30	3	Malawi
SC	F	Masters	26–30	1	Botswana
UJ	F	Masters	26–30	1	Zimbabwe
WA	F	Masters	26–30	1	Egypt
WE	F	Masters	26–30	1	Lesotho
XA	F	Masters	26–30	2	Egypt
ZN	F	Bachelor	21–25	1	South Africa
ZT	F	Masters	26–30	2	Tanzania

This table presents participant demographics including gender (15 females, 1 male), age range (21–30), academic qualifications (14 master’s degrees, 2 bachelor’s degrees), teaching experience (ranging from <1 to 3 years), and their teaching locations across 12 African countries. All participants were volunteer Chinese teachers in Confucius Institutes and Classrooms.

This study employed a two-round interview process with each participant. The initial round examined how teachers regulated their emotions and followed professional norms while interacting with various school stakeholders. Interviewees shared insights on emotional expectations in African educational environments and their contextual approaches to emotional management. Follow-up interviews were conducted to address unclear responses from initial conversations and obtain supplementary insights in the second round.

Each interview, conducted in Chinese to ensure nuanced communication between the native Chinese-speaking researcher and participants, was audio-recorded with participant consent and typically lasted 40–60 min. Interview recordings underwent transcription and English translation procedures. Data analysis employed MAXQDA software (2022) and thematic analysis techniques to identify recurring and co-occurring themes and interpret complex meanings within the data (Guest et al., 2012). A three-stage analytical process was adopted. Initially, an inductive approach was employed, grounded in the interview content. Subsequently, recognizing the relevance of emotional labor theory, a deductive approach guided further analysis (Braun and Clarke, 2006). During this stage, excerpts related to emotional rules as well as emotional labor strategies were extracted from the transcripts, and emergent themes were described, numbered, and coded. Finally, the analytical process culminated in an integrated framework capturing emotional rules and their associated labor strategies (see Table 2). This analytical framework facilitated systematic examination by providing a structured approach to data classification.

**Table 2:** Analysis of Emotional Rules and Emotional Labor strategies.

Emotional rules	Examples of emotional rules	Corresponding emotional labor strategies
1 Professional rules		
Conformity to professional teaching guidelines with emotional commitment	“I consciously strive to maintain an enthusiastic atmosphere” and “My job is to be patient and find ways to make learning accessible”	Surface Acting and Deep Acting
Adhering to the rules of the teaching profession in native culture	“All my strictness is for your own good” and “I criticised him for his own good because I expected more from him”	Pretending and Expressions of naturally felt emotions
Managing emotional challenges in professional competency	“become a teacher who cannot be stumped”	Disguising
2 Organizational rules		
Respect for hierarchy	“always insist on being polite (to the leader)” and “Who would confront them directly?”	Surface Acting and Deep Acting
Avoidance of conflict and sensitive topics	“avoid even glaring at students who misbehave” and “avoid discussing political issues”	Surface Acting
Deeply held commitment to cultural communication	“fostering a genuine sense of the interwoven nature of Chinese and African cultures”	Deep Acting
3 Socio-cultural rules		
Respect the local culture	“express our concern and then gently move on”	Cognitive Reappraisal
Conforms to the local impression of Chinese people	“consciously or unconsciously make yourself conform to this kind of humble image”	Surface Acting and Deep Acting
4 Personal rules		
Maintain a good relationship with students	“connect with each other on a personal level, like friends”	Deep Acting and Expressions of naturally felt emotions
Keep students motivated	“offering encouragement and praise is crucial”	Surface Acting and Expressions of naturally felt emotions

This table displays the thematic analysis results of emotional rules and corresponding emotional labor strategies. The analysis identified four main categories: Professional Rules, Organizational Rules, Socio-cultural Rules, and Personal Rules. Each category includes specific examples from participant interviews and the associated emotional labor strategies employed by teachers in their cross-cultural teaching contexts.

## 4 Findings

In this section, we identify and categorise four emotional rules, each of which contains a variety of emotional labour strategies elaborated from the interviews. According to the interview data, the emotional rules affecting the VCTs in their working life in Africa were professional rules, organisational rules, socio-cultural rules, and personal rules, in descending order.

### 4.1 Professional rules among VCTs

This section explores three key dimensions of professional emotional rules governing VCTs in African cross-cultural educational contexts. The analysis examines how Chinese language teachers, influenced by their home culture’s perceived professional emotional norms, navigate and emotionally invest in these rules in a different cultural environment. Additionally, it investigates how these emotional rules shape teachers’ deployment of various emotional labor strategies in their cross-cultural Chinese language teaching roles.

#### 4.1.1 Conformity to professional teaching guidelines with emotional commitment: “I consciously strive to maintain an enthusiastic atmosphere” and “my job is to be patient and find ways to make learning accessible”

As language educators, VCTs’ primary emotional guideline centers on encouraging active learner engagement with the target language. Given that speaking represents both an objective and essential process in language acquisition (Bashir et al., 2011), and considering Koran’s (2015) emphasis on creating anxiety-free learning environments, VCTs frequently employ emotional management strategies to foster positive classroom atmospheres. These teachers not only follow professional guidelines but also invest significant emotional energy to create effective learning environments. As Teacher AB describes:

A cheerful classroom is better than a silent or serious one. Silence is a taboo in ICCE classrooms, and learning a language means speaking more. In the classroom, students will be willing to open their mouths to speak Chinese as long as they are happy; as long as they practise, we will learn better and better…. I consciously strive to maintain an enthusiastic atmosphere in class. I actively engage students in discussions about Chinese culture, food and development, and listen to their perspectives about similar things in their country. The two-hour classes always pass quickly in such a pleasant environment. While maintaining a professional demeanor, I try to add my personal touch to make the learning experience more enjoyable (Teacher AB).

Similarly, Teacher LM recounts her approach to handling classroom challenges:

Teaching absolute beginners can be frustrating when they struggle with basic pronunciation day after day. But I tell myself, ‘These students come from completely different language backgrounds—it's not their fault. My job is to be patient and find ways to make learning accessible.’ I prepare mentally before each class with deep breathing exercises. Instead of drilling grammar rules, I design cultural activities like paper-cutting that incorporate Chinese characters. When I see their faces light up while creating art, I know this emotional investment is worth it. Professional guidelines say ‘be patient,’ but true teaching means adapting that patience to each student's needs (Teacher LM).

Rather than rigidly adhering to professional guidelines, these teachers demonstrate a combination of surface and deep emotional labor strategies. Teacher AB consciously manages her emotions to maintain an “enthusiastic atmosphere” while also genuinely investing herself in cultural exchanges. Likewise, Teacher LM employs surface acting (regulating frustration through breathing exercises) and deep acting (reframing challenges as opportunities for cultural connection), transforming the abstract rule of “patience” into concrete pedagogical practices. This dual approach to emotional labor—combining conscious emotion management with authentic engagement—creates what Liu et al. (2024) describe as an “anxiety-free” learning environment that prioritizes student enjoyment over traditional strict methods.

The cases of AB and LM collectively demonstrate how emotional rules and emotional labor strategies work together in cross-cultural teaching contexts. While AB exemplifies maintaining positive affect, LM showcases managing negative emotions—both teachers ultimately internalize professional guidelines as personal emotional commitments, going beyond mechanical compliance to achieve genuine educational outcomes.

#### 4.1.2 Adhering to the rules of the teaching profession in native culture: “all my strictness is for your own good!” and “I criticised him for his own good because I expected more from him”

A study suggests that many Chinese teachers deliberately exaggerate their emotional expressions in front of their students in order to create a certain atmosphere that will help them achieve specific goals. Most of the teachers interviewed in this study said that they usually show negative expressions when students misbehave and deliberately exaggerate the intensity of these expressions in order to ‘threaten’ the students, so that the students feel pressure to change their behavior (Yin, 2016). Therefore, Chinese teachers have never shied away from displaying negative emotions in front of their students, and they believe that displaying negative emotions is an effective way to “educate” their students. There is even a Chinese saying, “a knife’s mouth but a caring heart”, which describes this kind of harsh but caring behavior.

Most of the interviewed Chinese language teachers stationed in Africa knew very little about the local situation and culture before they went to African countries. Although they would receive pre-departure training from language cooperation centres or Confucius Institutes before they went to Africa, the content of the training did not go into detail about the culture and customs of each country, but more about the commonalities of the African countries, and even less about the rules of the profession of the teachers in African countries. However, the training did not cover the culture and customs of each country in detail. Therefore, when these VCTs go to the local communities to carry out teaching, they often do not know much about the local professional rules for teachers, and they carry out teaching activities with their own knowledge of the professional rules for teachers in the native culture, and they transfer the use of emotional labour strategies in the local teaching contexts according to the professional rules for teachers in the native culture. Therefore, the use of emotional labour strategies in the local teaching context is often encountered as a result of the impact between the teacher’s native culture and the local culture. For example, Teacher HW said:

In China, there is an old saying that ‘once a teacher, always a father’, and in the hearts of students, they also long for a ‘father’ or ‘mother’ from China. I believe that the majority of international Chinese teachers hold the belief that they should treat their students wholeheartedly… I believe that most international Chinese teachers have the belief that treating their students wholeheartedly is a form of parental love, so be bold and say in the classroom, ‘I am like a father to you, I want you to learn as much as you can, and all my strictness is for your own good!’ (Teacher HW)

Teacher HW’s words show that Chinese teachers try to transfer the teacher-student relationship and teachers’ “strict requirements” for students from their native clture to African Chinese teaching classrooms, trying to make African students understand and accept the intention behind the teacher’s “strict” image. This reveals that the Chinese international teachers’ intention is to care for the students’ growth and development behind the image of “strictness”. This passage shows that the VCTs’ knowledge of the professional rules for teachers is more based on their own native culture. But will this kind of transplantation which ignores the local culture go so smoothly? Let us look at the example of Teacher UJ:

Once, in my class, a few students did not listen attentively to the lesson, I was a bit angry, and when I saw that he (a certain student) was also talking with other students, I thought how could he be as disrespectful as the other students, so I named him on the spot, he explained that he was discussing learning problems with his classmates, but I didn't believe it at that time and criticised him anyway. He explained that he was discussing his studies with his classmates, but I didn't believe him and criticised him anyway. Since then, he became very depressed in my class and didn't take the initiative to answer my questions, which was very disappointing and saddening for me. I think he should understand that I criticised him for his own good because I expected more from him. (Teacher UJ)

In Teacher UJ’s statement, we can see that the teacher believes that the students’ talking in class is a form of disrespect for learning and for the teacher, so she takes a serious approach to criticising the students in public, regardless of their “face.” She thought that the students should understand that her criticism was for the sake of the students and because she had high expectations of them. Research reveals that Chinese educators interpret the expression of negative emotions in teaching through a distinct cultural lens. Their professional philosophy suggests that disciplinary actions or critical feedback reflect pedagogical commitment rather than personal aversion. This perspective aligns with traditional Chinese educational values, where instructional strictness represents a form of teacher care (Yin, 2016). So the student who had been scolded was expected to change their misbehavior instead of being angry with Teacher UJ. But instead, the student lost the initiative to learn and became negative from then on. It can be seen that the transfer of the rules of the Chinese teacher’s profession and the use of responsive emotional labour strategies did not always achieve the results expected by the Chinese teachers. This also reflects beginning teachers’ adaptation of professional norms and emotional management strategies in African educational contexts.

#### 4.1.3 Managing emotional challenges in professional competency: “become a teacher who cannot be stumped”

While the Standards for International Chinese Language Teachers (Hanban/Confucius Institute of China, 2012) specify language proficiency requirements, novice volunteer teachers, despite being native speakers, often experience emotional stress when facing advanced-level instruction due to limited teaching expertise and sometimes non-TCFL backgrounds. These challenges lead teachers to employ both surface and deep emotional labor strategies to maintain their professional image and develop their teaching competency. Teacher WE said.

The second difficulty for me is the high level of student demand on the teacher's ability to answer professional knowledge. k5 students are very hard working and smart, I really haven't seen such good students, so that means they will ask a lot of questions—about some word identification…, after I finish speaking, students will still ask all kinds of questions… So it requires the teacher to have a strong knowledge base ability to answer their questions. When I first started the class, I was a little bit flustered because the students will ask you questions from time to time, and I sometimes get stumped, but I don't show it, I think it's better to become a teacher who cannot be stumped, right? (Teacher WE)

While VCTs possess native speaker advantages, maintaining professional authority while pursuing educational objectives necessitates ongoing emotional labor. Through surface acting, they mask their uncertainty in challenging teaching moments, while through deep acting, they transform their professional anxiety into motivation for continuous development. As Schwimmer and Maxwell (2017) note, teachers must consistently enhance their professional skills for effective student education, a process that involves significant emotional investment and management.

### 4.2 Organisationally dominated emotional rules

Beyond professional guidelines, organizational frameworks significantly influence VCTs’ emotional practices. Three distinct organizational principles emerge: respect for hierarchy, avoidance of conflict and sensitive topics, deeply held commitment to cultural communication. These organizational elements constitute the second strongest determinant of emotional labor strategies within this African educational setting.

#### 4.2.1 Respect for hierarchy: “always insist on being polite (to the leader)” and “who would confront them directly?”

Analysis reveal that hierarchical respect is one of the principal causes, besides professional norms in teaching, why most teachers choose to conceal their true feelings. Almost all volunteer Chinese language teachers indicated that their work experiences at local Chinese language teaching institutions in Africa vary greatly depending on the management style and approach of their respective leaders. Volunteer Chinese language teachers in Africa have direct Chinese supervisors who manage them, eliminating the need for direct communication with leaders of the local African schools. As a result, organizational rules are more influenced by Chinese institutions rather than local African customs. In China, hierarchical respect is highly emphasized, especially in professional relationships, which significantly influences teachers’ emotional display in professional settings. For instance, the majority of novice teachers expressed that they might not agree with the school leaders’ instructions when communicating with them. However, they choose to maintain a smile and conceal their dissatisfaction to minimize organizational friction. The following statement from Teacher AB is a good example:

It's really about communicating directly with the administrator or the dean about the student's situation… But of course in the process of expressing ourselves, we will always insist on being polite (to the leader) anyways. (Teacher AB)

This example illustrates that even in everyday communication with their leaders, volunteer Chinese language teachers are mindful of their emotions and behavior, maintaining respect for their leaders’ authority. In another teacher’s description, we can see that even when teachers have clear dissatisfaction with their leaders, they still choose to suppress their negative emotions and maintain a calm and polite demeanor when communicating with them. Teacher SC said:

Although I don't particularly agree with some of the management styles and behaviors of the leaders, who (nobody) would confront them directly? Instead, I handle it with a more diplomatic approach.(Teacher SC)

These examples illustrate how volunteer Chinese language teachers in Africa navigate organizational hierarchies by suppressing their true emotions and avoiding direct conflicts with their superiors. They adopt emotional labor strategies to conform to the emotional regulations within their organizations.

#### 4.2.2 Avoidance of conflict and sensitive topics: “avoid even glaring at students who misbehave” and “avoid discussing political issues”

We also found that volunteer Chinese language teachers place great importance on harmonious relationships with their local colleagues and students in cross-cultural settings. Many of the interviewed teachers mentioned the principle of avoiding conflicts in cross-cultural environments, highlighting that this organizational rule is emphasized during the volunteer teachers’ selection and training process. As dispatched volunteer teachers from language cooperation centers/Confucius Institutes, they need to be particularly cautious in their words and actions to avoid straining the cooperative relationship between the two countries due to their own behavior. Teacher KC stated:

Because of significant cultural differences, language learning can be quite challenging. Furthermore, if we encounter uncontrollable situations in the classroom, I believe we must abide by local laws and regulations and avoid insulting, hitting, or even glaring at students who misbehave. These actions are inappropriate because, in a foreign country, we must first protect ourselves and, secondly, avoid engaging in such controversial behaviors.(Teacher KC)

The statement describes how teachers suppress and adjust their emotions in foreign environments to comply with organizational rules and avoid trouble. In addition, Chinese language volunteer teachers abroad are also expected to follow an unspoken rule: avoid discussing sensitive political topics with local colleagues or students, such as issues related to Hong Kong SAR or Taiwan region of China. For example, Teacher AB said:

When I was teaching in a different cultural context, I met a student who… He loved Chinese culture very much, so he was very happy to talk to me about some of China's history, including issues related to Taiwan. He spoke very well, and this was something I didn't expect at the time. Because when we teach, we may try our best to avoid discussing political issues. But when he took the initiative to talk to me, I would listen patiently… (Teacher AB)

This statement illustrates the cautious and tolerant attitude adopted by Chinese international volunteer teachers in Africa when faced with potentially controversial sensitive topics. The teacher may not be entirely comfortable with or agree with all of the student’s views, but he chooses to outwardly accept and listen in order to maintain classroom harmony and the student’s freedom of expression. He copes with this challenge by controlling his outward expressions, such as maintaining a calm facial expression and tone of voice.

#### 4.2.3 Deeply held commitment to cultural communication: “fostering a genuine sense of the interwoven nature of Chinese and African cultures”

Many VCTs demonstrated a profound identification with their role as cultural ambassadors, disseminating Chinese language and culture globally. While pre-departure training emphasizes this broader role, interviews revealed that interactions with African students significantly strengthened this sense of identity. The vast majority of volunteers, upon completing their one-year service, expressed pride in their mission, citing it as pivotal in shaping their career paths and inspiring them to continue teaching Chinese internationally. Furthermore, a substantial number expressed a desire to return to Africa to further their work in cultural exchange and language education. Teacher WE said:

As a dispatched teacher, I view teaching as more than just a duty to fulfill. My true focus lies in fostering a genuine sense within each African student learning Chinese of the interwoven nature of Chinese and African cultures. I strive to help them understand how the Belt and Road Initiative, a project of both Chinese and global significance, is deeply connected to their lives and how the vision of a shared future for humankind is inextricably linked to their own destiny. (Teacher WE)

This statement illustrates that international VCTs deeply resonate with and are dedicated to their educational content and mission. This profound identification and passion are cultivated through deep acting. Initially, this sense of mission is instilled through the training and dissemination activities organized by their institutions, which reinforce the volunteers’ comprehension and commitment to the mission. In practice, these volunteers do not merely adjust their expressions and behaviors superficially to match expected emotional displays. Instead, they engage in a deeper emotional adjustment to truly align their feelings with the intended expressions.

Deep acting not only enables teachers to synchronize their emotions with the organization’s mission expectations but also enhances the authenticity of their interactions, making their emotional expressions more natural and persuasive. At the same time, this authenticity deepens students’ engagement and understanding. Employing this strategy not only fosters a profound and positive teacher-student relationship but also significantly alleviates the emotional labor involved for the teacher, making their teaching experience more sustainable and fulfilling.

### 4.3 Socio-cultural regulated emotional rules

Beyond professional and organizational guidelines, socioculturally regulated emotional rules significantly shape Chinese volunteer teachers’ emotional labor strategies. Analysis reveals two principal socio-cultural patterns: adapting to host cultural norms and aligning with local expectations of Chinese cultural representatives. These proved to be important factors in influencing and guiding the teacher volunteers’ use of emotional labour strategies in this particular African context.

#### 4.3.1 Respect the local culture: “express our concern and then gently move on”

In a study, it was shown that the perception of tardiness varies from country to country, but in general Chinese people are quite punctual (Matondo, 2012). While it is common in Africa (e.g., Ethiopia, Botswana, Nigeria, Ghana) to hear about “African time,” which Ezekweslili (n.d) describes as“the perceived cultural tendency, in most parts of Africa, towards a more relaxed attitude to time”(Kgosi, 2016). According to Umez (2014) it is well known that an invitation card of a typical African event normally starts very late. In Africa punctuality is valued but not required; one can be early but will be kept waiting for some time before an event can start, or when one comes late it is acceptable even when they do not offer any explanation for their lateness (Foster, 2002). When people come late to any event. In the school setting, tardiness is also a common problem for both lecturer and students in Africa. This has become one of the most common problems encountered by VCTs teaching in Africa, where students are late or even absent without any explanation to the Chinese teachers before or after the class, which is sometimes considered “disrespectful to the teacher” from the perspective of the Chinese teachers. In this regard, the VCTs clearly demonstrated in the interviews that they had struggled with this, but almost all of them showed tolerance and understanding in the end. For example, Teacher ZT said:

In Africa, people do not have a strong sense of time, so in most cases, students are often not punctual. My advice is to ‘respect the customs’… Students have been living in their country for so many years that the weak sense of time and the lack of punctuality are already deeply rooted in their minds, and we should not try to change them overnight. When faced with this situation, we should first express our concern, then go on to express our understanding of the concept of time and the importance of punctuality. This will surely have some positive impact. I know that tardiness is very common in Africa and even if teachers try to emphasize it, they can't change it. It takes time to develop good behavior, so teachers don't have to be pushy, but it's a good idea to express our concern and then gently move on.(Teacher ZT)

This statement demonstrates the volunteer’s cultural sensitivity in navigating cultural differences while teaching. Faced with conflicting perspectives, the volunteer actively seeks to understand and adapt to the students’ cultural backgrounds, adjusting their own behaviors and expectations accordingly. This adaptation is not merely a personal choice but a crucial aspect of cultural sensitivity required for educators working in multicultural environments. It reflects a deep respect and understanding of local cultures.

#### 4.3.2 Conforms to the local impression of Chinese people: “consciously or unconsciously make yourself conform to this kind of humble image”

Our interviews indicated that VCTs, through their limited interactions with local communities in Africa, primarily with local colleagues, encountered various local perspectives on Chinese cultural characteristics. Among these cultural impressions, the perception of Chinese people as humble was the most frequently reported observation. Such an impression and evaluation more or less influenced the Chinese teachers in a foreign country. This expectation had a certain impact on VCTs, and some of them internalized it as an emotional rule. As Teacher SC said:

If you are in the local area, you will hear that the local people have the impression that ‘you Chinese are all very humble’, so you will be influenced by this assessment and will consciously or unconsciously make yourself conform to this kind of humble image. (Teacher SC)

From the expression of Teacher SC we can see that in Africa, due to the prevailing perception of Chinese people in the local culture, the individual teacher feels an external social pressure or expectation and adopts the strategy of adjusting his or her behavior to fit this perception. Behavioral adjustment in this situation is an adaptation to the socio-cultural situation, and through surface action strategies, individuals are able to exhibit behaviors that meet external expectations without changing their true inner feelings. This helps to reduce conflict and increase understanding and acceptance in multicultural communication.

### 4.4 Personal rules shaped by language teaching practice

Through Chinese language instruction, VCTs develop personal rules that reflect their unique pedagogical experiences. These personal rules create distinctive approaches, shaped by individual professional backgrounds and classroom encounters. These rules led them to use expressions of deeply performed or naturally felt emotions, such as “connect with each other on a personal level, like friends” and “offering encouragement and praise is crucial.”

#### 4.4.1 Maintain a good relationship with students: “connect with each other on a personal level, like friends”

The results of the interviews showed that more than half of the teachers agreed that they had an equal relationship with their students and were willing to maintain a good friendship with their students outside the classroom. Some teachers said they would accept students’ invitations to go to students’ homes or participate in important occasions such as students’ birthday parties and weddings after class. This kind of teacher-student relationship extended outside the classroom not only deepens the relationship between teachers and students and promotes students’ enthusiasm for learning on the students’ side, but also helps the teacher volunteers to gain a deeper understanding of the local culture and facilitates the teachers’ integration in their life and work. For example, Teacher LC stated:

In my opinion, the relationship between me and my students is not the traditional hierarchical teacher-student relationship, we can speak freely and discuss in class, and we can also chat and share outside of class, connect with each other on a personal level, like friends. This has won me the love of many students, who call me their ‘favourite Chinese teacher’. (Teacher LC)

It’s evident that the volunteer teachers prioritize genuine and natural interactions with their students. This emphasis on authenticity fosters deeper teacher-student relationships, creating a comfortable and respectful learning environment where students feel more empowered to actively participate and engage. This teaching style exemplifies a student-centered approach, utilizing natural emotional expression to strengthen the bond and communication between teachers and students. This method not only benefits students’ academic growth but also cultivates their fondness and trust in the teacher, further demonstrating the significance of human connection and empathy in the educational process.

#### 4.4.2 Keep students motivated: “offering encouragement and praise is crucial”

According to findings, almost all VCTs reported that they actively gave positive encouragement and praise to their students when they showed progress or saw their students making efforts to learn. This process shows the teachers’ genuine feelings. Some teachers also capitalize on this motivational effect by making extensive use of positive feedback such as encouragement and praise between teacher-student interactions to motivate and promote students’ enthusiasm for the teacher as well as for learning. For example, Teacher QB said:

Using emotional connection as a foundation for education is a really sound approach. It's about making sure students not only enjoy the class itself but also feel a positive connection with the teacher. But how do we achieve that? … One way is for teachers to express genuine fondness and appreciation for their students. Offering encouragement and praise is crucial—‘You did a fantastic job!’, ‘I'm so proud of your effort!’, ‘That sentence is beautifully written!’, ‘Your homework is incredibly thorough!’ Everyone appreciates kind words and encouragement, and a teacher who offers them generously will undoubtedly be well-loved." (Teacher QB)

The aforementioned example illustratese that Teacher QB may need to express a positive reaction that is stronger than what he actually feel inside at certain moments in order to encourage and motivate their students. Although this expression may not fully reflect the teacher’s true emotions, it is intended to create a more supportive and motivating learning environment.

## 5 Discussion

The feeling rules identified in this study both align with and extend previous research on volunteer teacher emotional labor. While Afreen and Norton (2024) identified general expectations for heritage language volunteer teachers, our findings reveal that VCTs face additional culturally-specific emotional rules, particularly those related to cross-cultural adaptation and cultural ambassadorship roles in African contexts. This suggests that the institutional context and cultural positioning of volunteer teachers significantly shapes the specific emotional rules they encounter. Examining emotional labor practices of VCTs in African contexts enhances our understanding of cross-cultural language instruction. This study extends emotional labor theory by demonstrating how professional, organizational, sociocultural, and personal emotional rules interact in cross-cultural teaching contexts. The findings suggest that emotional rules are not merely external constraints but are actively interpreted and internalized by teachers through their professional practice. This dynamic process is particularly evident in how VCTs navigate between their native cultural understanding of teaching and local educational norms, creating a unique pattern of emotional labor that combines surface acting, deep acting, and naturally felt emotions. Furthermore, the study reveals how novice teachers in cross-cultural contexts develop their emotional competence through continuous negotiation between different layers of emotional rules.

Our findings indicate that teachers’ emotional labor strategies are influenced by four regulatory frameworks: professional, organizational, sociocultural, and personal dimensions. These emotional parameters typically function as tacit, subtle influences. The emotional labor strategies adopted by the volunteer teachers included surface acting, deep acting, and expressions of naturally felt emotions, through which teachers applied various techniques such as pretending, disguising, and cognitive restructuring.

Professional norms include language teaching codes, cultural teaching guidelines, and language proficiency standards, each requiring significant emotional commitment. As Loh and Liew (2016) note, language teaching demands diverse emotional investments. VCTs not only follow professional guidelines but actively invest emotional energy to create effective learning environments, employing both surface and deep acting strategies. While surface acting helps maintain professional demeanor in challenging situations, deep acting enables teachers to transform their professional anxiety into authentic engagement with students. Despite training, novice teachers may struggle with cultural understanding and professional competency, leading to complex emotional labor strategies. Hulda and Zhao (2024) recommend culturally sensitive training for novice teachers, suggesting pre-departure preparation in local educational dynamics to reduce unnecessary emotional labor.

Beyond professional emotional rules, organizational norms exert a significant influence on VCTs. While the governing Chinese institutions (namely Confucius Institutes and language centers) rarely assert strong pressure, teachers recognize an unwritten norm of maintaining politeness in their interactions with supervisors. During moments of disagreement, these educators frequently choose to either distance themselves from conflict or engage in apparently logical discourse rather than express their true concerns. Furthermore, teachers are strongly advised to avoid conflicts with locals and refrain from discussing sensitive political topics. These organizational norms are emphasized during the volunteer selection and training process and are strictly upheld by local managing institutions in Africa. Consequently, when students bring up sensitive subjects in class, teachers often choose to listen patiently without extensive commentary, opting to either steer clear of the topic or redirect the conversation back to language learning. Therefore, when navigating these organizational emotional rules, teachers actively employ both surface acting and deep acting strategies. Research has also revealed that the majority of VCTs have internalized their role as ambassadors of Chinese culture, as defined by their organizations. They employ deep acting strategies to meet the organizational requirements associated with this role. As noted by Akin (2021), “Internalized teacher commitment may be an important factor in sustaining teachers’ abilities to align their emotional displays with school expectations.” This suggests that a deeply rooted sense of duty significantly influences how effectively teachers can adhere to the emotional norms prescribed by their educational organizations.

Our study also sheds light on the role of African sociocultural values in shaping emotional rules for volunteer Chinese language teachers. These rules include respecting local culture and being mindful of local perceptions and expectations of Chinese people. In adhering to these sociocultural norms, teachers engage in both surface acting and deep acting. In Africa, the perception of punctuality differs from that in China. This often leads to students arriving late, being absent, or even providing no explanation for their absence. This difference in time orientation poses a direct challenge to volunteer teachers’ teaching schedules and is a significant cultural difference experienced by many. However, upon understanding the cultural context, teachers often choose to acknowledge these differences and engage in rational communication with students, demonstrating a deep acting strategy of cognitive reappraisal. Moreover, while interacting with local colleagues and community members, teachers are also influenced by local perceptions of Chinese people. This phenomenon aligns with the self-fulfilling prophecy. Rosenthal and Jacobson's (1968) research, while focused exclusively on target-based expectations (i.e., those derived from an individual’s personal attributes), suggests a broader implication: just as expectations based on individual traits can create self-fulfilling prophecies, so too can stereotype-based expectations (Ashmore and Del Boca, 1981), which stem from generalized beliefs about social groups. When individuals hold stereotypes about a group, their interactions with members of that group may elicit behaviors that inadvertently confirm those pre-existing beliefs. Local African perceptions of Chinese people can create sociocultural pressures on VCTs, requiring them to employ emotional regulation strategies to navigate these expectations.

In line with Hulda and Zhao's (2024) findings, this study further confirms the influence of personal rules, rooted in individual traits and beliefs, on emotional labor strategies. This reinforces the understanding that individual characteristics play a significant role in shaping how individuals manage their emotional displays in the workplace. These personal rules are self-constructed and implemented during their teaching and interactions with students. Some are designed to ignite students’ passion for learning Chinese, while others aim to boost their motivation and engagement. Throughout this process, we observed these teachers employing various emotional labor strategies, with a particular emphasis on the expression of naturally felt emotions. Research shows that volunteer Chinese language teachers in Africa demonstrate a high level of emotional regulation.

While this study relied primarily on interview data, future research could benefit from multiple data sources including classroom observations and teacher reflective journals to provide more comprehensive insights into VCTs’ emotional labor experiences. The limited recent literature on volunteer language teachers’ emotional labor highlights the need for more contemporary research in this area.

## 6 Conclusion

This study examines emotional labor strategies among volunteer Chinese teachers dispatched to Africa by Confucius Institutes/language cooperation centers and investigates the emotional rules influencing their strategic choices. Our findings reveal a hierarchical influence on teachers’ emotional labor: professional norms exert the strongest influence, requiring both conformity to guidelines and significant emotional commitment. This is followed by organizational norms, sociocultural norms, and personal rules. Understanding these rules facilitates teachers’ adaptation to local environments, enabling appropriate professional identity projection and effective interpersonal communication. These findings illuminate how teachers combine surface and deep acting strategies to meet professional requirements while maintaining authentic emotional engagement.

Furthermore, the study also reveals that novice VCTs experience varying levels of stress stemming from their professional roles and requirements, the organizational structure, and the challenges of adapting to a foreign culture. These teachers frequently employ strategies to mask or regulate their true emotions, engaging in significant emotional labor. This bidirectional nature of cross-cultural teaching challenges is evident when compared with Jackson and Adarlo's (2016) study, where American volunteers in China struggled with examination-focused teaching and large class sizes, while our Chinese VCTs faced different but equally challenging adaptations in African contexts, particularly regarding time orientation and communication styles. Moreover, our findings indicate that these novice teachers often internalize and manage stress independently, lacking external or organizational support, which poses a significant challenge to their mental and emotional well-being. This finding aligns with Zhang (2021), who observed that volunteer teachers experience “cyclical instability” in their emotions and must rely heavily on “self-agency” to regulate their emotional states, suggesting that inadequate institutional support is a broader challenge in volunteer teaching contexts. Therefore, providing training and support related to emotional labor is crucial for enhancing their overall well-being. We strongly recommend implementing regular emotional training programs designed to equip these teachers with coping mechanisms for stress management, emotional regulation, and resilience building. These programs should incorporate evidence-based practices, such as mindfulness techniques, stress reduction strategies, and interpersonal communication skills training. In addition, we advocate for ongoing psychological support services, readily accessible and tailored to the specific needs of volunteer teachers. By providing these comprehensive resources, volunteer teachers will be better equipped to navigate the emotional demands of their work, ultimately leading to improved job satisfaction, reduced burnout, and enhanced overall well-being.

## Data Availability

The interview data that support the findings of this study are not publicly available due to participant confidentiality agreements. Further inquiries about the data can be directed to the corresponding author, and access may be considered upon reasonable request with appropriate ethical approval.
